# Intranasal oxytocin blunts amygdala response to negative affective stimuli in males and females with alcohol use disorder: a randomized controlled cross-over trial

**DOI:** 10.1007/s00213-025-06779-x

**Published:** 2025-03-31

**Authors:** Sina Vetter, Sophia Schnabel, Matthias Reichl, Lea Sirignano, Valery Grinevich, Anne Koopmann, Rainer Spanagel, Falk Kiefer, Wolfgang Sommer, Patrick Bach

**Affiliations:** 1 Department of Addictive Behavior and Addiction Medicine, Medical Faculty Mannheim, Central Institute of Mental Health, University of Heidelberg, Mannheim, Germany; 2 German Center for Mental Health (DZPG) - Partner Site Mannheim-Heidelberg-Ulm, Mannheim, Germany; 3 Feuerlein Center on Translational Addiction Medicine (FCTS), University of Heidelberg, Mannheim, Germany; 4 Department of Genetic Epidemiology in Psychiatry, Medical Faculty Mannheim, Central Institute of Mental Health, University of Heidelberg, Mannheim, Germany; 5 Department of Neuropeptide Research in Psychiatry, Medical Faculty Mannheim, Central Institute of Mental Health, University of Heidelberg, J5, 68159 Mannheim, Germany; 6 Institute of Psychopharmacology, Medical Faculty Mannheim, Central Institute of Mental Health, University of Heidelberg, Mannheim, Germany; 7 Bethanien Hospital for Psychiatry, Greifswald, Germany

**Keywords:** Alcohol use disorder, Negative affect, Oxytocin, Amygdala, Faces-task, fMRI

## Abstract

**Rationale:**

Negative affect plays a prominent role in the maintenance of alcohol use disorder (AUD) and has been identified as a risk factor for relapse to alcohol. To date, however, treatment options that target negative affective states and consecutive relapse risk in AUD are insufficient. Oxytocin (OXY) might be a promising approach for addressing negative affective states and resulting motivation to use alcohol.

**Objectives:**

We aimed to investigate the acute effects of 24 I.U. OXY, administered intranasally, compared to matched placebo (PLC) on central processing of negative emotional stimuli in the amygdala in individuals with AUD.

**Methods:**

We conducted a randomized double-blind placebo-controlled crossover study in *N* = 24 individuals with AUD. Amygdala response to emotional stimuli served as primary outcome and was assessed using a validated functional magnetic resonance imaging emotion-processing task. Alcohol craving served as secondary outcome.

**Results:**

OXY versus PLC attenuated right amygdala reactivity to fearful and angry emotional face stimuli during the fMRI task (*t*(33) = 3.32, *p*_FWE_=0.035), while no effect of OXY on amygdala activation was observed during the presentation of geometric figures. In addition, right amygdala reactivity to fearful and angry emotional face stimuli was positively associated with alcohol craving (*r* =.332, Bias corrected and accelerated 95% confidence interval [95% BCa CI]=-0.044 to 0.624, *p* =.042).

**Conclusions:**

OXY’s effects on the neurocircuitry underlying negative affect and craving in AUD support its potential for dampening alcohol craving induced by negative affective states and implicate OXY as a potential future treatment option for AUD.

**Clinicaltrials registry:**

DRKS00026218.

**Supplementary Information:**

The online version contains supplementary material available at 10.1007/s00213-025-06779-x.

## Introduction

Despite the high prevalence of AUD worldwide and its tremendous impact on global health (World Health Organization 2018), only a few medications are approved for its treatment that overall show only limited efficacy (Litten et al. 2018; Rosner et al. 2010). Especially negative affective states, which have been identified as a major driving force of excessive goal-directed drug choice and risk factor for exacerbated craving and relapse risk in AUD (Heilig et al. 2019; Oslin et al. 2009; Witkiewitz and Villarroel 2009), are only insufficiently addressed by approved medications. Previous research indicated that negative affect and relapse to alcohol are dynamically linked, suggesting that targeting negative affect in AUD could decrease craving and relapse risk (Oslin et al. 2009; Witkiewitz and Villarroel 2009). Still, effective treatment options are lacking. In this context, oxytocin (OXY), an endogenous neuropeptide, might be a potential candidate, due to its effects on emotion processing neurocircuits (Grinevich and Neumann 2021; Quintana and Guastella 2020). Specifically, the amygdala has been identified as a brain region relevant for the processing of negative affective states in addiction (Koob and Volkow 2010), in particular with respect to the oxytocinergic system (Koob 2021; Koob and Vendruscolo 2023). Within the amygdala, a substantial number of oxytocinergic fibers and oxytocin receptors have been localized (Gimpl and Fahrenholz 2001), suggesting OXY is a potential mediator of the responses to negative emotional stimuli and occurrence of negative affective states. Indeed, direct manipulation of OXY receptors and OXY levels within the amygdala resulted in altered affective states and reduced distress in animal models (Bale et al. 2001; Ebner et al. 2005).

Preclinical studies reported that OXY attenuated anxious behavior and alcohol use in rats (Bowen et al. 2011) and that application of OXY reduced self-administration of alcohol in mice (King et al. 2017), prairie voles (Potretzke et al. 2023; Stevenson et al. 2017) and rats (Hansson et al. 2018; MacFadyen et al. 2016). These effects are centrally mediated. In this context, a recent animal study demonstrated significant effects of OXY on alcohol-induced GABA release in the central nucleus of the amygdala and a corresponding reduction of alcohol consumption (Tunstall et al. 2019).

In humans, effects of OXY on alcohol-related phenotypes are heterogenous. A pilot study in alcohol dependent patients reported a reduction of withdrawal symptoms, after administration of 24 IU OXY per day for three days (Pedersen et al. 2013) and we found reduced reactivity to alcohol cues in the insular cortex, the hippocampus / parahippocampal gyrus, the cingulate gyrus, the inferior and medial frontal gyrus, and in visual and motor regions after a single administration of 24 IU OXY (Hansson et al. 2018). On the other hand, a following study failed to replicate these findings (Melby et al. 2019) and a recent randomized controlled trial showed no significant effects of OXY (40 IU up to three times per day) versus placebo on alcohol consumption, relapse risk and craving, thus questioning direct effects of OXY on these phenotypes (Melby et al. 2021). Still, the latter trial indicated significant effects of OXY on subjective nervousness, pointing towards a potential effect on negative affective states. The idea that OXY’s effects in AUD might be indirect, via effects on emotion processing and the underlying neurocircuits, is also in line with another clinical trial that showed a significant moderation of OXY’s effects by attachment anxiety. For example, OXY reduced craving only in subjects with higher attachment anxiety (Mitchell et al. 2016). Effects of OXY on the amygdala, as a central hub of emotion-processing neurocircuits, were consistently reported across a large number of studies that used an emotion-processing task to probe the response of the amygdala to emotional stimuli (Domes et al. 2007; Kirsch et al. 2005; Tully et al. 2018), supporting the general potential of OXY to modulate emotion-processing neurocircuits. Regarding the effects of OXY in populations that regularly use alcohol, a pilot study by our group in male social drinkers could show that OXY attenuated amygdala response to negative face expressions, which was associated with lower subjective alcohol craving and a lower percentage of heavy drinking days (Bach et al. 2021). However, OXY effects on emotion-processing neurocircuits and craving in individuals with AUD has so far not been studied. To address this question, we conducted a randomized placebo-controlled crossover study in males and females with AUD to investigate the acute effects of OXY on amygdala response to emotional stimuli and alcohol craving using a validated functional magnetic resonance imaging emotion processing paradigm. The single-dose design enabled us to examine the immediate effects of OXY within a randomized-controlled crossover design, providing insights into whether OXY could be used as an acute, on-demand medication during high craving states resulting from negative affective states. We hypothesized that OXY reduces amygdala activation during processing of negative emotional stimuli, and that higher amygdala activation during processing of negative emotional stimuli is associated with higher subjective alcohol craving after the cue-exposure, i.e. before the imaging session, and higher subjective alcohol craving after the imaging session.

## Methods

The project (Target-OXY) as a whole was designed to investigate the effects of OXY compared to PLC on experimental models for two central stages of the addiction cycle, specifically striatal alcohol cue-reactivity and amygdala response to emotional stimuli that were chosen, due to their close links with alcohol craving. Results on the alcohol cue-reactivity paradigm represent a different pre-registered analysis that are reported elsewhere (Vetter et al. 2025). In short, we found that OXY compared to PLC increased alcohol cue-induced brain activation in parts of the frontal gyrus, supplementary motor area, hippocampus, parts of the temporal gyrus, fusiform gyrus, parahippocampal gyrus, precuneus, the superior parietal gyrus (*p*_FWE_<0.05 whole-brain cluster-level corrected). We found no significant effect of OXY in the ventral striatum and OXY increased cue-induced craving that was measured via visual analogue scales during the fMRI alcohol cue-reactivity task. However, we observed a significant interaction of treatment x sex on cue-induced craving during the fMRI alcohol cue-reactivity task. Female participants reported higher while males reported lower cue-induced alcohol craving during fMRI after OXY compared to PLC application.

Here we focus on the analyses of OXY’s acute effects on emotion processing, i.e. amygdala response to emotional stimuli, and its association with alcohol craving measured via the Alcohol Urge Questionnaire (AUQ) after cue-exposure with the favorite alcoholic drink, i.e. directly before fMRI measurement (AUQ second assessment), and after fMRI session (AUQ third assessment).

### Study design

We conducted a randomized, double-blind, placebo-controlled crossover study (clinical trial registry: DRKS00026218) to investigate the effects of intranasal OXY on amygdala activation during the presentation of fearful and angry face stimuli in 24 individuals with AUD. After screening and enrolment, individuals were randomized to one of two assessment sequences (OXY-PLC, PLC-OXY) to control for sequence effects. The two study visits were scheduled at an interval of 7 to 14 days, to reduce risk of any carryover effects. Participants received OXY either at the first or at the second study visit and a matched PLC during the other visit, according to their sequence group. Matched PLC sprays were identical in odor and appearance, containing the same ingredients except for the active ingredient Oxytocin. Both nasal sprays were blinded and packaged in identical brown glass bottles with a spray nozzle by the study pharmacy. Neither study personnel nor participants were able to notice any differences between OXY and PLC. A single dose of 24 IU OXY (Syntocinon, Oxytocin, ATC code: H01BB02) was administered intranasally (6 puffs in each nostril) 45 min prior to the functional magnetic resonance imaging (fMRI) session. The intranasal application, the dose and timing were chosen in line with previous studies, which reported a maximum effect of OXY on brain response and behaviour between 20 and 90 min after intranasal application (Quintana et al. 2021).

### Study sample

In total, 139 non-treatment seeking participants with AUD that took part in a national cohort study, as part of the German collaborative research center TRR265 (Heinz et al. 2020; Spanagel et al. 2024), were re-contacted and screened for inclusion and exclusion criteria after completing the observational study. Of those, 24 individuals with AUD, according to DSM-V (at least two AUD criteria fulfilled and exclusion of individuals with withdrawal symptoms), between 18 and 65 years were eligible for participating in the trial and have provided written informed consent (see Supplementary Table S1). In addition, participants needed to have normal vision. Contraindications for MRI, pregnancy, lactation and breastfeeding, as well as severe somatic (Long QT-syndrome or other severe heart disease, liver cirrhosis) or psychiatric comorbidities (psychotic disorders, mania, bipolar disorder, severe depression with suicidal ideation) were exclusion criteria and assessed by trained study personnel via structured assessment of the medical history. Intake of psychotropic medication (except antidepressants from the group of serotonin re-uptake inhibitors or mirtazapine and pipamperone), was not permitted. Positive urine drug screening (amphetamines, opiates, benzodiazepine and cocaine) or breath alcohol > 0.0‰ at either one of the two treatment visits led to exclusion from the study.

**Fig. 1:**
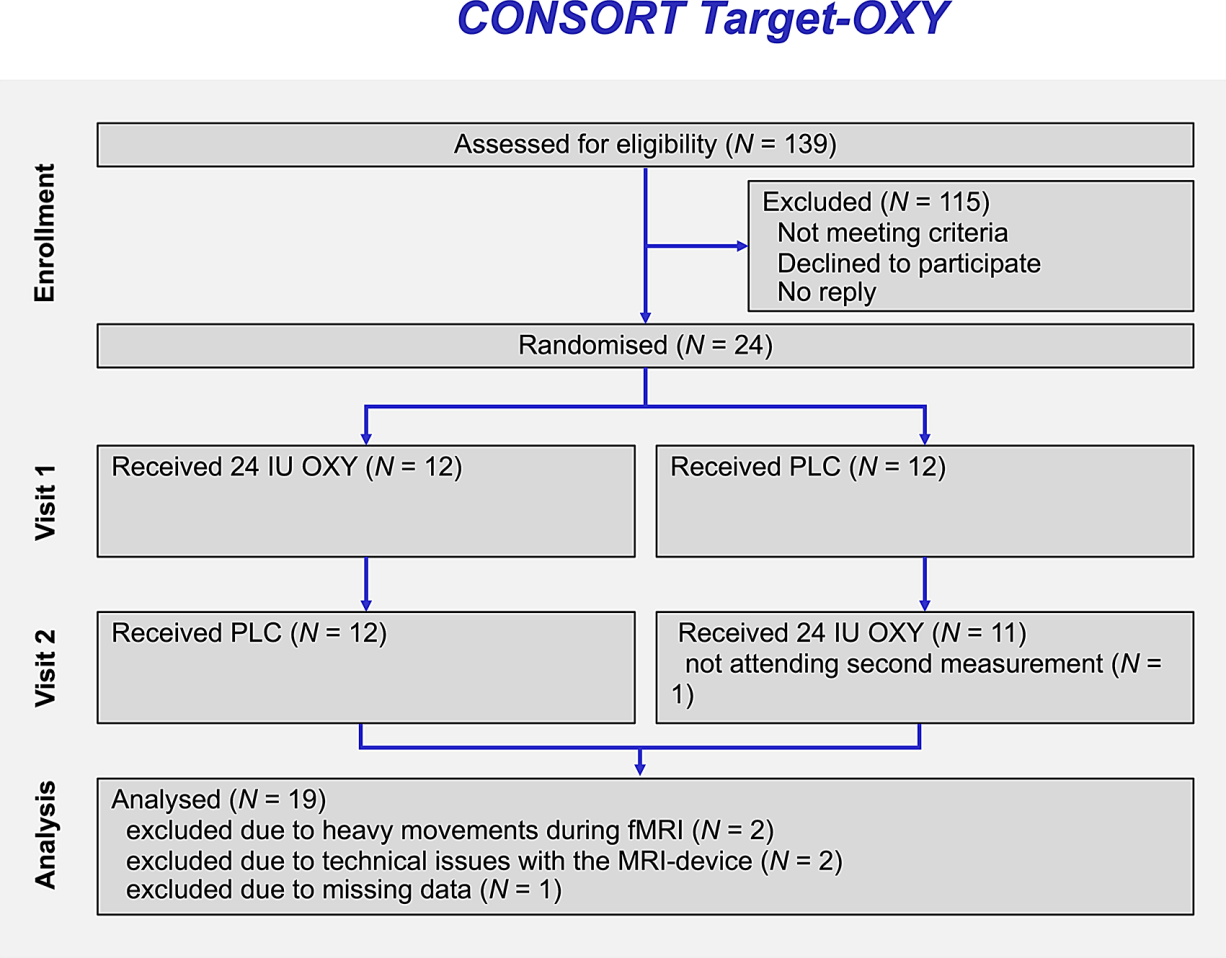
CONSORT-Flowchart

Of the 24 participants, one did not attend the second scanning session (drop out), and four participants had to be excluded from further analysis due to heavy movement during the fMRI session or technical issues with the MRI device (see CONSORT flow chart, Fig. 1). According to statistical analysis plan, only complete datasets were considered in the analysis of the primary endpoint. In total datasets of 19 participants were analysed.

### Study procedure

**Fig. 2:**
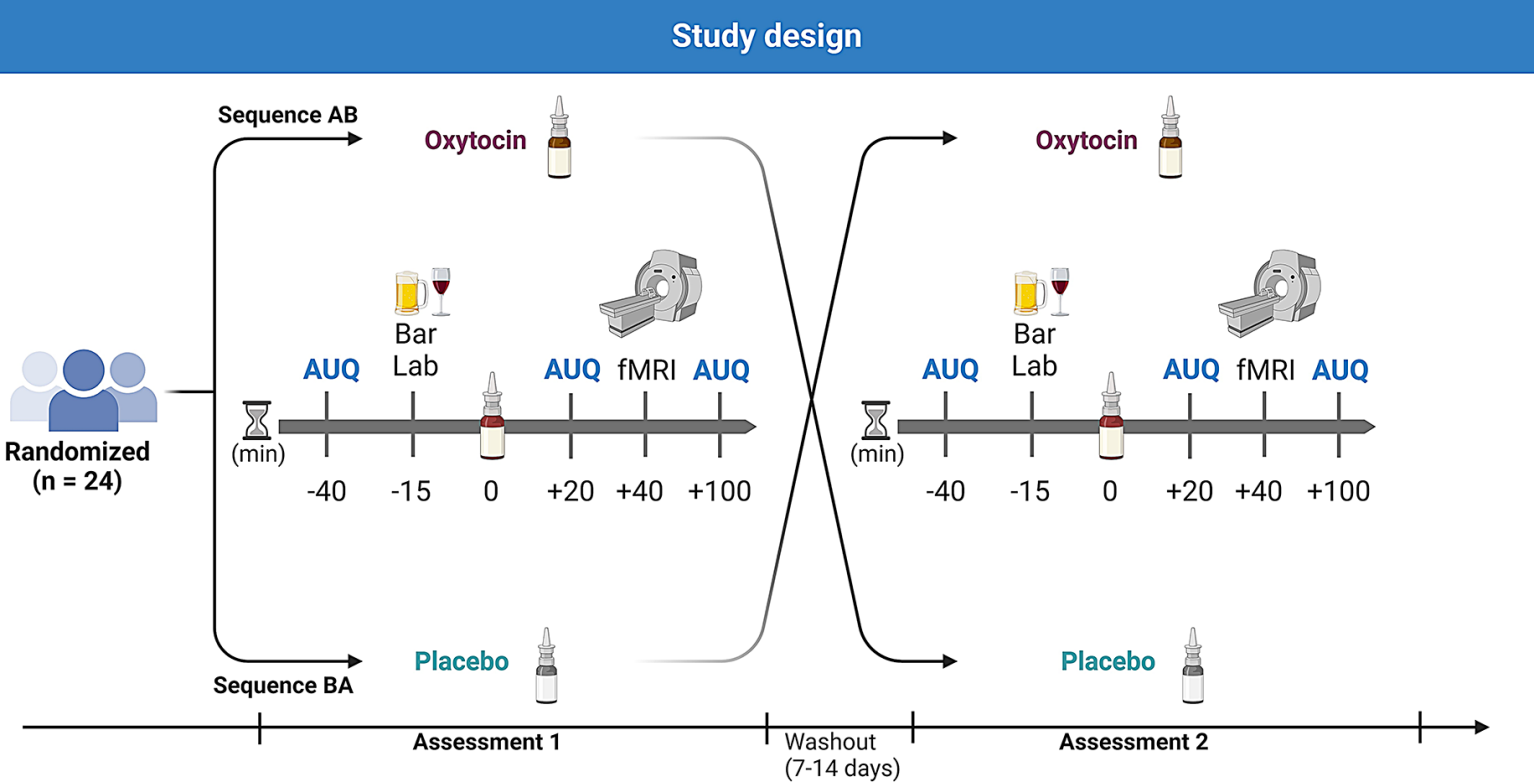
Depiction of the study design and procedures. Either 24 I.U. Oxytocin (OXY) or Placebo (PLC) were administered intranasally after an alcohol cue-exposure in a bar lab setting. 40 min after administration of OXY or PLC an emotion processing face-matching task was performed during functional magnetic resonance imaging (fMRI) to investigate Amygdala response. Alcohol craving was measured at three timepoints by using the Alcohol Urge Questionnaire (AUQ). Created with BioRender.com

Apart from the treatment with OXY or PLC, both study visits followed the same procedures. Each study visit started with a verification of the absence of any exclusion criteria. To this end, participants underwent drug urine screening, breath alcohol test, and in women with childbearing potential, a pregnancy test was performed. Afterwards, baseline alcohol craving was assessed using the Alcohol Urge Questionnaire (AUQ; (Bohn MJ 1995). Further, the Obsessive Compulsive Drinking Scale (OCDS-G; (Mann 2000), the Beck Depression inventory (BDI-II; (Hautzinger 2009), the State-Trait-Anxiety inventory (STAI; (Laux et al. 1981) and the Perceived Stress Scale (PSS; (Cohen et al. 1994) were administered to assess craving in the last days, depressive symptoms, anxiety and subjective stress levels as baseline characteristics. Alcohol cue exposure with the preferred alcoholic beverage was conducted in a laboratory bar setting for three minutes in line with an established procedure (Kwako et al. 2015; Monti et al. 1987) to induce alcohol craving directly before OXY or PLC administration. Twenty minutes after intranasal application of OXY or PLC, a second measurement of the AUQ, was completed. Afterwards participants were prepared for fMRI measurement, then the alcohol cue-reactivity task (Vollstadt-Klein et al. 2012) started, followed by the face matching task (Hariri et al. 2002), a resting state measurement and a structural measurement. Participants underwent a third measurement of the AUQ after completing MRI. Study procedures are depicted in Fig. 2.

The ethics committee of the medical faculty Mannheim of the University of Heidelberg approved all experimental procedures (AZ 2021 − 515).

### Primary and secondary endpoints

The parent study was designed to investigate the effects of OXY compared to PLC on alcohol cue-reactivity and Amygdala response to emotional stimuli. Here we focus on the amygdala response to emotional stimuli in a validated fMRI emotion-processing task measured by the blood oxygenated level dependent (BOLD) response, that was defined as primary outcome in this analysis. Situational alcohol craving measured by the AUQ (second and third assessment, directly before and after the fMRI) and performance data i.e., accuracy and reaction times during fMRI served as secondary endpoint. For the here presented analyses, the AUQ score following the alcohol exposure in the Bar Lab setting (second AUQ assessment) was of particular interest as a secondary endpoint, as it represents situational craving in an ecologically valid drinking scenario, which may be relevant for the context of craving triggered by negative affective states.

### fMRI emotion processing face-matching task

During fMRI all participants performed a face-matching task adapted from Hariri and colleagues (Hariri et al. 2002). The task consisted of 4 shape- and 4 faces-blocks with 6 trials per block. In each block either shapes or faces with angry or fearful expressions were presented. Participants had to decide whether the left or right face or shape at the bottom matches with the face or shape at the top of the screen. They were instructed to choose the matching face or shape by pressing the left or right button on a MRI-compatible response pad. Reaction times and accuracy (correct vs. incorrect button presses) were recorded during the task using the Presentation^®^ software (Version 16.0, Neurobehavioral Systems Inc., Albany, CA, USA). The task took approximately five minutes in total.

### Assessment and pre-processing of fMRI data

The fMRI measurements were conducted using a Siemens MAGNETOM 3 Tesla whole-body-tomograph (MAGNETOM PRISMA^fit^, Siemens, Erlangen, Germany). During the faces task a total of 306 T2*-weighted echo-planar images (EPI) were acquired during the alcohol cue-reactivity task using the CMRR multi-band EPI sequence(Moeller et al. 2010; Setsompop et al. 2012) (TR = 0.869 s, TE = 38 ms, flip angle = 58°, 60 interleaved slices, slice thickness = 2.4 mm, voxel dimensions = 2.4 × 2.4 × 2.4 mm^3^, FOV = 210 × 210mm^2^, 88 × 88 matrix, AP phase-encoding, multi-band factor 6, bandwidth 1832 Hz/Px, MB LeakBlock Kernel, weak raw filter, prescan normalization, excite pulse duration 7ms). Field map images were acquired with a standard Siemens dual gradient echo sequence (TR = 0.698 s, TE1 = 5.19 ms, TE2 = 7.65 ms, flip angle = 54°, 64 interleaved slices, slice thickness = 2.4 mm, voxel dimensions 2.4 × 2.4 × 2.4 mm^3^, FOV = 210 × 210mm^2^, 88 × 88 matrix, AP phase-encoding, bandwidth 279 Hz/Px).The first five scans were excluded from further analyses, in order to reduce artifacts resulting from magnetic saturation effects. All MRI data were pre-processed using the statistical parametric mapping software for Matlab (SPM, version 12, Wellcome Department of Cognitive Neurology, London, UK). MRI data were temporally realigned and corrected for residual geometric distortion on the basis of the magnetic field map, spatially realigned and corrected for micromovements and normalized to a standard MNI (Montreal Neurological Institute, Quebec, Canada) EPI template. Following this step, an isotropic Gaussian kernel for group analysis (8 mm full width at half maximum) was applied to the images.

### Statistical analyses

Analyses of the neuroimaging data were performed according to previous studies, by firstly modelling the experimental conditions (faces vs. shapes) of the fMRI task in a generalized linear model (GLM) using the statistical parametric mapping software for Matlab (SPM, version 12, Wellcome Department of Cognitive Neurology, London, UK) to compute the first-level contrast images that contrast brain activation during the task conditions against implicit baseline (contrast: faces– baseline, shapes– baseline) and against another (contrast: faces– shapes). According to the crossover design, a flexible factorial model with the factors treatment (OXY vs. PLC), period (OXY at first vs. OXY at second measurement) and subject was set up in SPM12 and first-level contrast images (see above) were included to test the treatment effects of OXY versus PLC on amygdala activation. Treatment effects on amygdala activation during the presentation of emotional face stimuli and shapes were assessed combined and separately, to confirm that the effects were specific to the emotion-processing condition. The carryover effect was evaluated by including the period-by-treatment interaction in the flexible factorial model (Lim and In 2021). To control for sex effects, sex (male vs. female) was included as covariate. Due to the strong a priori hypothesis for OXY’s effects on the amygdala, a region-of-interest (ROI) approach was used to determine the significance of OXY effects on the amygdala response to emotional stimuli. To this end, a standardized anatomical amygdala mask from the Wake Forest University PickAtlas was used to define the left and right amygdala ROI (WFU PickAtlas, version 3.0.2, https://www.nitrc.org/projects/wfu_pickatlas). Significance was set to a local small-volume-corrected a family-wise error rate (FWE) corrected p value of p_FWE_<0.05. To complement the analyses of averaged brain activation, we also extracted block-wise activation values from the left and right Amygdala (betas) using the region of interest mask for the left Amygdala and the right Amygdala and the MarsBaR toolbox (version 0.45, https://marsbar-toolbox.github.io/index.html) for Matlab. According to Wellek and Blettner (2012), the hypothesis of negligible carryover effect was initially tested by performing a t-test for independent samples for the intraindividual sums of the betas of sequence group AB (OXY-PLC) and sequence group BA (PLC-OXY). Subsequently, the intraindividual differences of the betas from sequence group AB (OXY-PLC) and sequence group BA (PLC-OXY) for faces blocks and shapes blocks were compared separately using t-tests for independent samples to test the specificity of the treatment effect. The analysis was performed using IBM SPSS Statistics (version 27.0). Additional post-hoc power analyses for the t-tests for independent samples of the intraindividual differences of the betas in the left and right amygdala for faces blocks were performed using G*Power (version 3.1.9.7) (Faul et al. 2009). Intervention effects on alcohol craving (AUQ before and after fMRI) and performance data i.e., accuracy and reaction times during face matching task were analysed using mixed models with treatment (OXY vs. PLC), sequence (OXY at first vs. OXY at second measurement) and sex (male vs. female) as fixed effects, and subjects as random effect in IBM SPSS Statistics (version 27.0). The period-by-treatment interaction was included in the mixed model analysis to account for potential carryover effects. In addition, baseline characteristics were compared to control for sequence effects or any systematic differences between the sessions. Statistical significance level was set to α = 0.05.

To investigate the hypothesized association between amygdala activation and alcohol craving, we extracted functional brain activation in the left and right amygdala ROI using the MarsBaR software package (version 0.45, https://marsbar-toolbox.github.io/index.html) and imported the values into IBM SPSS Statistics (version 27.0). Associations of functional brain activation in the left and right amygdala ROIs with craving (AUQ) were tested using Pearson correlation coefficient and robustness was confirmed using bootstrapping (i.e. the Bias corrected and accelerated [BCa] bootstrapping procedure).

## Results

### Sample characteristics

In total, 19 (9 males) individuals with AUD (mean age = 45.63 ± 11.69) were included in the primary analyses with no significant differences in baseline characteristics between the OXY and PLC assessment sessions (see Tables 1 and 2).

**Table 1:** Demographic data and substance use patterns

	Individuals with AUD *N* = 19
*Demographical variables*	
Sex (female/male)	10/9
Age [years; mean (SD)]	45.63 (11.69)
*Substance use characteristics*	
Number of AUD criteria last 12 months	3.26 (2.21)
ADS [total score; mean (SD)]	7.47 (4.29)
Smoker (yes/no)	3/16
FTND^#^ [total score; mean (SD)]	1.37 (2.77)

Note. AUD = Alcohol Use Disorder, ADS = Alcohol Dependence Scale, FTND = Fagerstrøm Test for Nicotine Dependence, ^#^ assessed in smokers only

**Table 2:** Depiction of the baseline characteristics, alcohol craving and performance data for the sample of individuals with mild to severe alcohol use disorder for both treatment visits

Individuals with AUD (*N* = 19)	Mean (SD)	Mean (SD)	Statistics	*p*-value
**Baseline chracteristics**	**OXY**	**PLC**		
OCDS (sumscore)	9.47 (5.72)	9.16 (6.00)	*F*(1,17) = 0.60	*p* =.451
STAI (state sumscore)	38.05 (9.49)	38.00 (10.15)	*F*(1,17) = 0.01	*p* =.906
PSS (sumscore)	17.84 (6.22)	17.05 (5.36)	*F*(1,17) = 0.53	*p* =.477
BDI-II (sumscore)	9.79 (6.04)	9.21 (6.40)	*F*(1,17) = 0.23	*p* =.640
**Alcohol craving**	**OXY**	**PLC**		
AUQ_baseline_ (sumscore)	13.74 (5.85)	13.37 (5.79)	*F*(1,17) = 1.80	*p* =.677
AUQ_preMRI_ (sumscore)	14.42 (5.93)	16.21 (7.89)	*F*(1,17) = 1.73	*p* =.206
AUQ_postMRI_ (sumscore)	16.42 (7.97)	16.74 (9.02)	*F*(1,17) = 0.78	*p* =.784
**Performance data**	**Oxytocin**	**Placebo**		
Accuracy during **faces** conditions (%)	99.78 (0.96)	99.12 (2.23)	*F*(1,17) = 1.14	*p* =.301
Reaction time during **faces** task (ms)	1170.41 (226.95)	1210.48 (258.95)	*F*(1,17) = 1.24	*p* =.280
Accuracy during **shapes** condition (%)	96.93 (4.13)	96.05 (4.70)	*F*(1,17) = 0.29	*p* =.597
Reaction time during **shapes** task (ms)	1196.43 (276.04)	1198.83 (252.27)	*F*(1,17) = 0.32	*p* =.582

Note. AUD = Alcohol Use Disorder, OXY = Oxytocin, PLC = Placebo, OCDS-G = Obsessive Compulsive Drinking Scale, STAI = State and Trait Anxiety Inventory, PSS = Perceived Stress Scale, BDI-II = Beck Depression Inventory

The whole-brain analysis of the main effects of task conditions confirmed that face stimuli induced higher brain activation than shapes in a cluster of brain areas including the fusiform gyrus, the occipital gyrus, lingual gyrus and calcarine (*p*_FWE_<0.05 whole-brain cluster-level corrected) and in the left and right amygdala (*p*_FWE_<0.05 SVC-corrected). Higher amygdala activation was only observed for the face-matching condition, but not in the shapes condition, indicating the specificity of amygdala activation for face stimuli (see Table 3).

**Table 3:** Brain regions that show significant condition- (faces vs. shapes)-dependent activation (*p*_FWE_ < 0.05 whole-brain cluster-level corrected)

Side	Brain regions	Cluster size	MNI Coordinates			*t*_max_
Faces > Shapes						
R	Fusiform Gyrus, Inferior Occipital Gyrus, Lingual Gyrus, Middle Occipital Gyrus, Calcarine, Superior Occipital Gyrus, Cuneus	4123	-30	-86	-10	11.19
R	Amygdala^#^	79	20	-4	-16	5.64
L	Fusiform Gyrus, Middle Occipital Gyrus, Inferior Occipital Gyrus, Lingual Gyrus, Calcarine, Superior Occipital Gyrus	4123	-30	-86	-10	11.19
L	Amygdala^#^	86	-20	-6	-16	6.73
Faces < Shapes						
-						

Note. *t*_max_ = maximum t-value; *p*_*FWE*_ <0.05; ^#^= Region of interest (ROI) based small volume corrected (SVC) analysis

### Effects of OXY on emotion processing in the amygdala

**Fig. 3:**
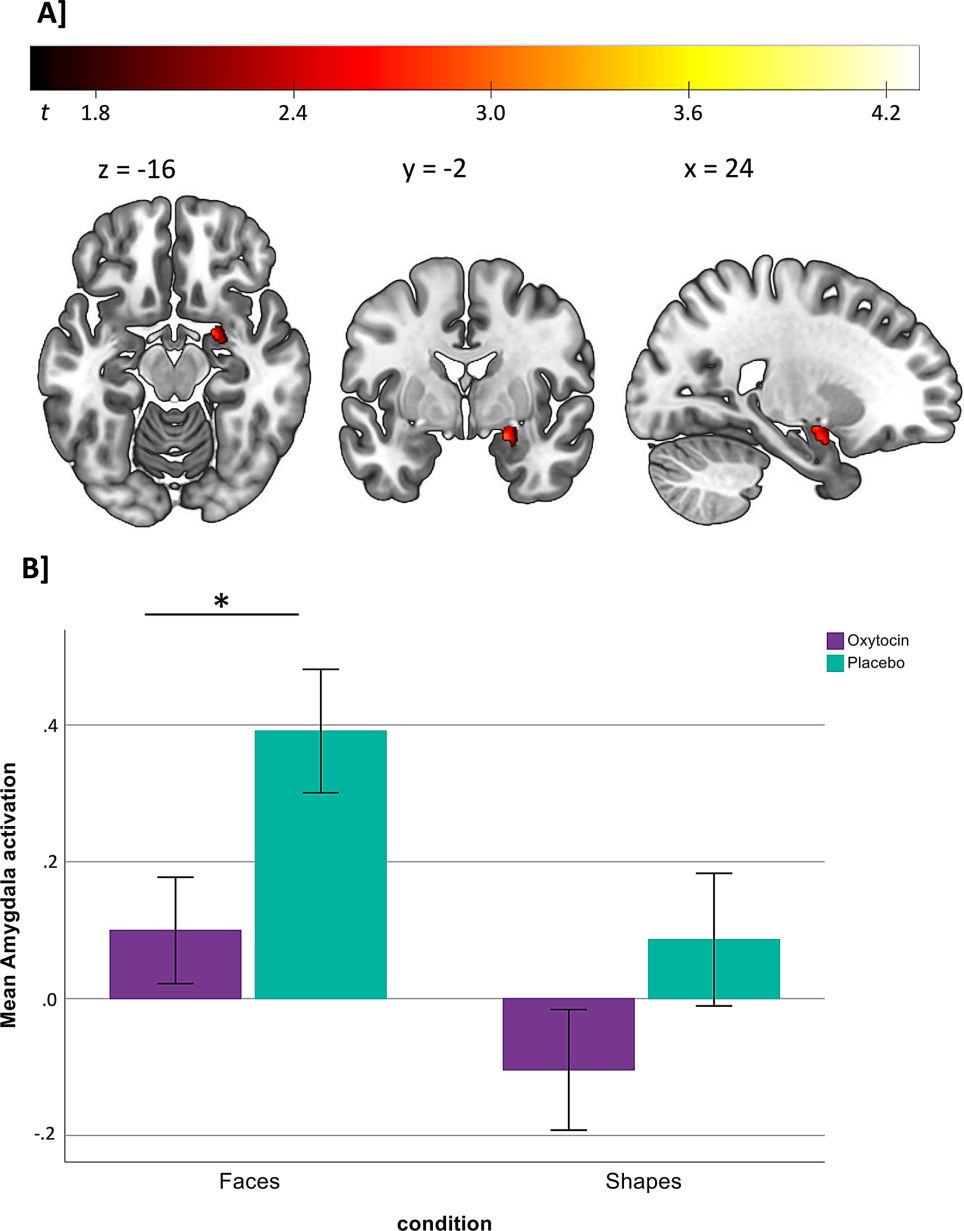
Depiction of the activation cluster in the right Amygdala, which showed a significant treatment effect with significantly lower activation after Oxytocin (OXY) compared to Placebo (PLC) administration (*p*_*FWE*_ < 0.05 small volume corrected for the Amygdala as pre-specified region of interest) and B] the difference in mean brain activation in the right Amygdala between OXY and PLC administration in the faces (*t*_*faces*_(18)=-2.609, *p* =.018, Cohen’s d = − 0.599) and shapes (*t*_*shapes*_(18)=-1.574, *p* =.133, Cohen’s d = − 0.361) blocks (as part of a post hoc test, mean activation for the faces and shapes blocks was extracted using the region of interest mask for the right Amygdala and the marsbar toolbox for Matlab; error bars represent +/- 1 standard error)

Our analyses of local activation in the amygdala showed that OXY significantly attenuated the response to emotional face stimuli (first-level contrast: faces - baseline) in the right amygdala (*t*(33) = 3.32, *p*_FWE SVC_ = 0.035, see Fig. 3), while no significant OXY effect was found on activation in the left amygdala. No significant OXY effect was observed during the presentation of geometric figures (first-level contrast: shapes - baseline) in the left or right amygdala. In addition, post-hoc analyses of the extracted mean activation for the faces and shapes blocks showed negligibility of carryover effects and that OXY versus PLC attenuated activation in the right amygdala during face-matching blocks (*t*_faces(17)_=-2.447, *p* =.026, Cohen’s d = -1.137) but not during shape-matching blocks (*t*_shapes(17)_=-1.362, *p* =.191, Cohen’s d = − 0.633) blocks (see Fig. 3), indicating that the effect of OXY was specific to the processing of emotional stimuli while the attenuated activation of OXY versus PLC during the face-matching blocks was not observed in the left amygdala (*t*_faces(17)_=-0.444, *p* =.662, Cohen’s d = − 0.206). A post-hoc power analysis was conducted to evaluate the statistical power of the t-tests for independent samples of the faces-blocks in the left and right amygdala. Based on the effect size observed in our data and the alpha level of 0.05, the analysis indicated that the achieved power was 76% for detecting differences between OXY and PLC in the right amygdala and 11% for detecting differences between OXY and PLC in the left amygdala. Given the observed effect sizes, the power analysis suggests that the sample size was not sufficient to detect the effects of small size in the left amygdala. Additional whole-brain analysis revealed no significant treatment effects (OXY vs. PLC) in brain areas beyond the amygdala during the presentation of emotional face stimuli.

To confirm that the observed differences in amygdala responses were not due to effects of OXY versus PLC on task performance, we compared reaction times between both treatment conditions. Here, we observed no significant difference between OXY versus PLC sessions concerning reaction times (*F*_shapes(1,17)_ = 0.315, *p* =.582, eta^2^ = 0.018; *F*_faces(1,17)_ = 1.243, *p* =.280, eta^2^ = 0.068) or accuracy (*F*_shapes(1,17)_ = 0.290, *p* =.597, eta^2^ = 0.017; *F*_faces(1,17)_ = 1.138, *p* =.301, eta^2^ = 0.063) during the face- and shape-matching trials of the fMRI task (see Table 2).

### Effects of OXY on alcohol craving

There was no significant effect of OXY versus PLC on situational alcohol craving at second (*F*_(1, 17)_ = 1.730, *p* =.206, eta^2^ = 0.092) and third assessment (*F*_(1, 17)_ = 0.078, *p* =.784, eta^2^ = 0.005) of the AUQ questionnaire after OXY or PLC administration during the experimental session.

### Associations between amygdala activation and behavioural data

**Fig. 4:**
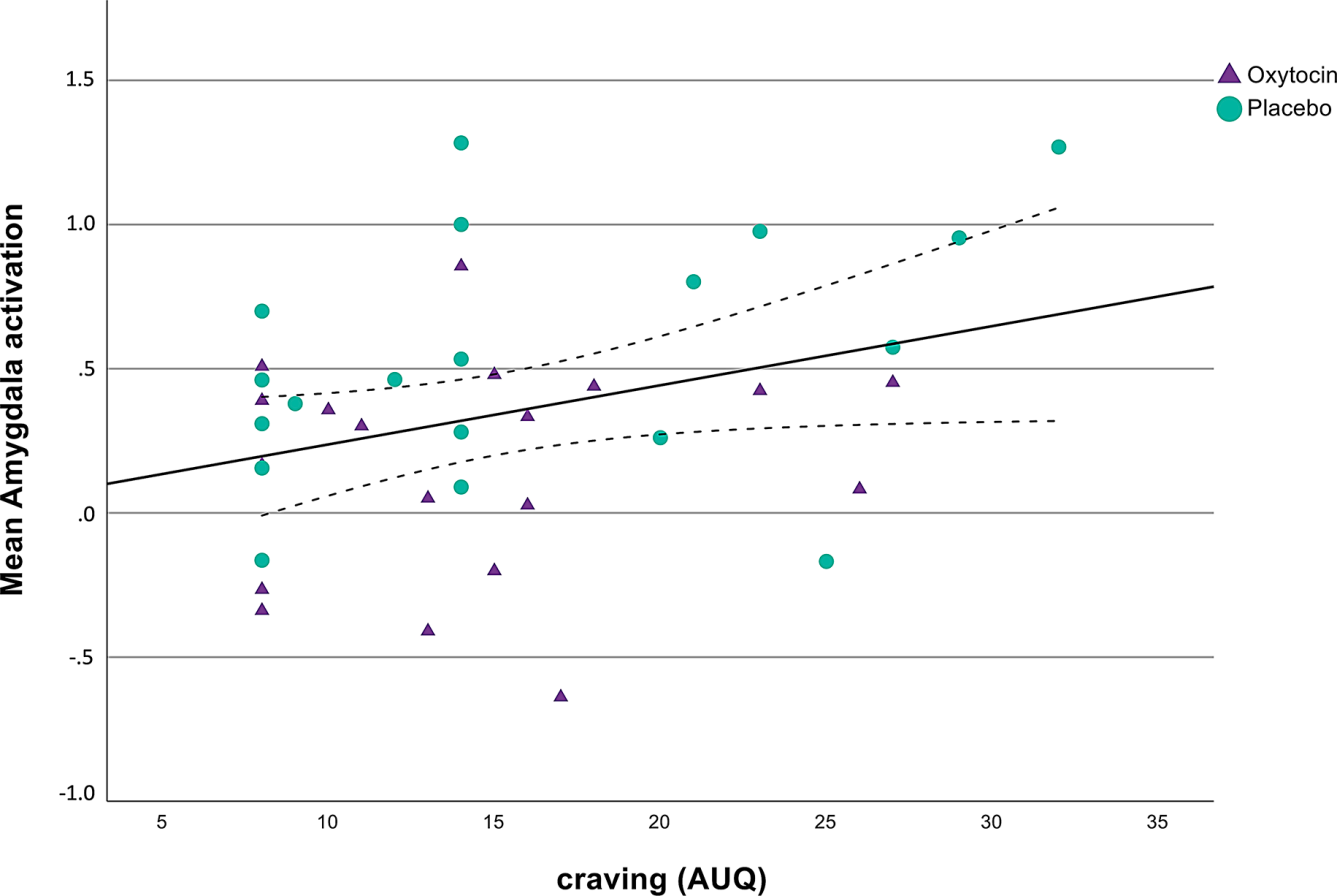
Depiction of the significant positive correlation between activation in the right amygdala and alcohol craving after treatment with oxytocin or placebo (second assessment of the Alcohol Urge Questionnaire (AUQ); *r* =.332, Bias corrected and accelerated 95% confidence interval [95% BCa CI]=-0.044 to 0.624, *p* =.042)

Right amygdala activation during the presentation of emotional face stimuli was significantly associated with situational alcohol craving after treatment with OXY or PLC and after cue-exposure with the favorite alcoholic drink which was measured directly before fMRI (second AUQ assessment; *r* =.332, Bias corrected and accelerated 95% confidence interval [95% BCa CI]=-0.044 to 0.624, *p* =.042; see Fig. 4 and Supplementary Figure S1), but the association with situational alcohol craving after fMRI measurement failed to yield significance (third AUQ assessment; *r* =.303, 95% BCa CI=-0.015 to 0.556, *p* =.064).

## Discussion

The results of this randomized double-blind placebo-controlled crossover study show that a single dose of OXY significantly attenuates amygdala activation during emotion processing, which in turn positively correlated with situational alcohol craving after cue-exposure with the favorite alcoholic drink. However, no direct treatment effect of OXY on situational alcohol craving after cue-exposure with the favorite alcoholic drink was observed. These findings suggest that OXY has significant effects on emotion-processing neurocircuits in AUD, which were implicated in goal-directed drug choice during negative affective states (Giannone et al. 2024; Hogarth 2020). However, the effect of OXY on craving might depend on the presence of negative affective states, indicating the context-dependence of OXY’s effects on alcohol craving with stronger effects when experiencing negative affective states. In accordance to previous research, which demonstrated a close link between negative affect and alcohol craving (Bresin et al. 2018; Suzuki et al. 2020), we observed a significant association between the amygdala response to fearful and angry face expressions and situational alcohol craving after cue-exposure with the favorite alcoholic drink, suggesting a higher sensitivity of the amygdala to emotional stimuli in individuals reporting higher situational alcohol craving after alcohol cue-exposure in the Bar Lab setting. Previous findings of our research group in social drinkers and independent studies in healthy individuals mirror presented findings (Bach et al. 2021; Domes et al. 2007; Kirsch et al. 2005), suggesting a robust effect of OXY an amygdala activation during the processing of emotional stimuli and also substantiating the association between amygdala activation and alcohol craving (Bach et al. 2021). Further studies also established direct links between amygdala activation and alcohol craving, supporting the role of emotion-processing neurocircuits in AUD and craving (Childress et al. 1999; Garrison et al. 2023; Schacht et al. 2013). In the light of these findings, presented results indicate that our prior findings in male social drinkers generalize to males and females with AUD, supporting the potential of OXY for treating craving induced by negative affective states.

Previous studies indicated that OXY’s effects might be different in males and females, even though findings are inconsistent (Hansson and Spanagel 2020; Lieberz et al. 2020; Potretzke et al. 2023). The presented trial was not designed to compare effects between sexes, but instead intended to recruit a balanced number of female and male individuals and control for sex in the analysis to allow generalizability of the effects of OXY effects in AUD for both biological sexes. In addition, this trial is designed as crossover design, which allows to control for inter-individual characteristics.

According to previous work, which showed that OXY effects on alcohol craving are linked to negative affect or more specific attachment anxiety (Mitchell et al. 2016), one could speculate that OXY might be especially effective in individuals experiencing negative affective states and anxiety through attenuation of dysbalanced amygdala activation. Further, the observed OXY effects on the BOLD response are unlikely to be performance effects, rather, they are more likely associated with the emotional component as OXY affects emotional processing without altering task performance during fMRI. However, OXY does affect the negative emotional states resulting from emotion processing, which in turn are related to craving. This might also explain why OXY is especially effective in individuals experiencing anxiety and negative affect. Even though presented results replicate previous findings and thus substantiate the robustness of OXY’s effects in AUD, future larger studies are needed to confirm OXY’s effects and determine whether effects attenuate after repeated OXY exposure.

### Limitations

The here presented study included a well-characterized sample of male and female individuals with mostly mild to moderate AUD. Thus, the results might not generalize to individuals with severe AUD, who typically suffer from higher craving states (Hansson et al. 2018). The success of the blinding was not systematically assessed, however, due to the matching of the OXY and PLC nasal spray and the blinding procedure no differences were reported by the study personnel or participants. Future studies should consider to assess the success of blinding systematically, which could further strengthen the validity of the results. In this trial, a balanced number of female and male individuals was enrolled and we controlled for sex in the analysis to allow generalizability of the effects of OXY in AUD for both biological sexes. Fluctuations of progesterone levels throughout the menstrual cycle might be associated with altered amygdala response and altered sensitivity for fearful faces in females (Derntl et al. 2008; Domes et al. 2010). However, the menstrual cycle or contraception was not assessed and therefore, not specifically considered in the analyses. The analyses did not reveal sex-effects, but it cannot be ruled out that the menstrual cycle in females may have affected the data. Additionally, due to delays of the study start caused by the COVID-19 pandemic, optimization of the MRI device, and regulatory requirements, which led to an adaptation of the study design and recruitment procedure where only participants from a well-characterized cohort were recruited, resulting in a reduction of the pool of eligible individuals, of whom some were no longer interested in participating in another study, the sample size differed from the originally planned sample size. The final sample size has limited the capacity to detect smaller effects, in particular in the left amygdala. Therefore, we have performed a post-hoc power analysis to evaluate the power of the effect of OXY versus PLC during faces-blocks in the left and right amygdala, which revealed that the sample size was not sufficient to detect effects of small size in the left amygdala. This suggests that potential effects in the left amygdala might have remained undetected in the presented study, due to the small sample size. While some studies observed effects on facial emotions only in the left amygdala, other studies found effects only in the right amygdala or in the bilateral amygdalae (Tully et al. 2018). There is some evidence for lateralization of effects, and the effects of sex and implicit/explicit paradigms have been discussed. However, more research is needed to further investigate lateralization. Our data do not directly support the lateralization hypothesis, as the effect in the left and right amygdala had at least descriptively the same direction, although the effect was not significant in the left amygdala. Still, the present study is the largest neuroimaging trial to investigate OXY’s effects in AUD so far, and power was increased through the crossover design.

Further studies are needed to confirm that the effects of OXY on neural circuits translate to clinical endpoints, such as craving and relapse over longer treatment periods. To this end, it is still unclear which schedule and dose of OXY is optimal for repeated administration over longer periods.

## Conclusion

The presented results provide evidence that a single-dose of OXY selectively and significantly attenuates amygdala activation during processing of negative emotional stimuli in individuals with AUD, supporting the effects of OXY on emotion-processing circuits underlying negative affective states, craving and relapse in AUD. The significant association between amygdala response to emotional stimuli and situational alcohol craving indicate the potential of OXY to relief craving-related pathology in AUD during negative affective states.

## Electronic supplementary material

Below is the link to the electronic supplementary material.

Supplementary Material 1

## Data Availability

The data that support the findings of this study are available upon request from the corresponding author, SV. The data are not publicly available due to the sensitive nature of the data that could compromise the privacy of research participants.

## References

[CR1] Bach P, Koopmann A, Bumb JM, Zimmermann S, Buhler S, Reinhard I, Witt SH, Rietschel M, Vollstadt-Klein S, Kiefer F (2020) Oxytocin attenuates neural response to emotional faces in social drinkers: an fMRI study. Eur Arch Psychiatry Clin Neurosci 271:873–88 32076819 10.1007/s00406-020-01115-0 PMC8236029

[CR2] Bale TL, Davis AM, Auger AP, Dorsa DM, McCarthy MM (2001) CNS region-specific Oxytocin receptor expression: importance in regulation of anxiety and sex behavior. J Neurosci 21:2546–2552 11264328 10.1523/JNEUROSCI.21-07-02546.2001 PMC6762393

[CR3] Bohn MJKD, Staehler BB (1995) Development and initial validation of a measure of drinking urges in abstinent alcoholics. Alcohol Clin Exp Res 19:600–606 7573780 10.1111/j.1530-0277.1995.tb01554.x

[CR4] Bowen MT, Carson DS, Spiro A, Arnold JC, McGregor IS (2011) Adolescent Oxytocin exposure causes persistent reductions in anxiety and alcohol consumption and enhances sociability in rats. PLoS ONE 6:e27237 22110618 10.1371/journal.pone.0027237 PMC3217952

[CR5] Bresin K, Mekawi Y, Verona E (2018) The effect of laboratory manipulations of negative affect on alcohol craving and use: A meta-analysis. Psychol Addict Behav 32:617 30010350 10.1037/adb0000383 PMC6136957

[CR6] Childress AR, Mozley PD, McElgin W, Fitzgerald J, Reivich M, O’Brien CP (1999) Limbic activation during cue-induced cocaine craving. Am J Psychiatry 156:11–18 9892292 10.1176/ajp.156.1.11 PMC2820826

[CR7] Cohen S, Kamarck T, Mermelstein R (1994) Perceived stress scale. Measuring stress: a guide for health and social scientists. 235–283

[CR8] Derntl B, Windischberger C, Robinson S, Lamplmayr E, Kryspin-Exner I, Gur RC, Moser E, Habel U (2008) Facial emotion recognition and amygdala activation are associated with menstrual cycle phase. Psychoneuroendocrinology 33:1031–1040 18675521 10.1016/j.psyneuen.2008.04.014 PMC7437605

[CR9] Domes G, Heinrichs M, Glascher J, Buchel C, Braus DF, Herpertz SC (2007) Oxytocin attenuates amygdala responses to emotional faces regardless of Valence. Biol Psychiatry 62:1187–1190 17617382 10.1016/j.biopsych.2007.03.025

[CR10] Domes G, Lischke A, Berger C, Grossmann A, Hauenstein K, Heinrichs M, Herpertz SC (2010) Effects of intranasal Oxytocin on emotional face processing in women. Psychoneuroendocrinology 35:83–93 19632787 10.1016/j.psyneuen.2009.06.016

[CR11] Ebner K, Bosch OJ, Krömer SA, Singewald N, Neumann ID (2005) Release of Oxytocin in the rat central amygdala modulates Stress-Coping behavior and the release of excitatory amino acids. Neuropsychopharmacology: Official Publication Am Coll Neuropsychopharmacol 30:223–230 10.1038/sj.npp.1300607 15536493

[CR12] Faul F, Erdfelder E, Buchner A, Lang A-G (2009) Statistical power analyses using G* power 3.1: tests for correlation and regression analyses. Behav Res Methods 41:1149–1160 19897823 10.3758/BRM.41.4.1149

[CR13] Garrison KA, Sinha R, Potenza MN, Gao S, Liang Q, Lacadie C, Scheinost D (2023) Transdiagnostic Connectome-Based prediction of craving. Am J Psychiatry 180:445–453 36987598 10.1176/appi.ajp.21121207

[CR14] Giannone F, Ebrahimi C, Endrass T, Hansson A, Schlagenhauf F, Sommer W (2024) Bad habits–good goals? Meta-analysis and translation of the habit construct to alcoholism. Translational Psychiatry 14:298 39030169 10.1038/s41398-024-02965-1 PMC11271507

[CR15] Gimpl G, Fahrenholz F (2001) The Oxytocin receptor system: structure, function, and regulation. Physiol Rev 81:629–683 11274341 10.1152/physrev.2001.81.2.629

[CR16] Grinevich V, Neumann ID (2021) Brain Oxytocin: how puzzle stones from animal studies translate into psychiatry. Mol Psychiatry 26:265–279 32514104 10.1038/s41380-020-0802-9 PMC7278240

[CR18] Hansson AC, Spanagel R (2020) No changes in the Oxytocin system in alcohol-dependent female rodents and humans: towards a sex-specific psychopharmacology in alcoholism. Addict Biol 26:e12945–e12945 32761675 10.1111/adb.12945

[CR17] Hansson AC, Koopmann A, Uhrig S, Buhler S, Domi E, Kiessling E, Ciccocioppo R, Froemke RC, Grinevich V, Kiefer F, Sommer WH, Vollstadt-Klein S, Spanagel R (2018) Oxytocin reduces alcohol Cue-Reactivity in alcohol-Dependent rats and humans. Neuropsychopharmacology 43:1235–1246 29090683 10.1038/npp.2017.257 PMC5916348

[CR19] Hariri AR, Tessitore A, Mattay VS, Fera F, Weinberger DR (2002) The amygdala response to emotional stimuli: a comparison of faces and scenes. NeuroImage 17:317–323 12482086 10.1006/nimg.2002.1179

[CR20] Hautzinger MK, Kühner F, Beck C, A.T (2009) Beck Depressions-Inventar: BDI II. Pearson Assessment, Frankfurt am Main, Frankfurt am Main

[CR21] Heilig M, Augier E, Pfarr S, Sommer WH (2019) Developing neuroscience-based treatments for alcohol addiction: A matter of choice? Translational Psychiatry 9:255 31594920 10.1038/s41398-019-0591-6 PMC6783461

[CR22] Heinz A, Kiefer F, Smolka MN, Endrass T, Beste C, Beck A, Liu S, Genauck A, Romund L, Banaschewski T, Bermpohl F, Deserno L, Dolan RJ, Durstewitz D, Ebner-Priemer U, Flor H, Hansson AC, Heim C, Hermann D, Kiebel S, Kirsch P, Kirschbaum C, Koppe G, Marxen M, Meyer-Lindenberg A, Nagel WE, Noori HR, Pilhatsch M, Priller J, Rietschel M, Romanczuk-Seiferth N, Schlagenhauf F, Sommer WH, Stallkamp J, Strohle A, Stock AK, Winterer G, Winter C, Walter H, Witt S, Vollstadt-Klein S, Rapp MA, Tost H, Spanagel R (2020) Addiction research consortium: losing and regaining control over drug intake (ReCoDe)-From trajectories to mechanisms and interventions. Addict Biol 25:e12866 31859437 10.1111/adb.12866

[CR23] Hogarth L (2020) Addiction is driven by excessive goal-directed drug choice under negative affect: translational critique of habit and compulsion theory. Neuropsychopharmacology 45:720–735 31905368 10.1038/s41386-020-0600-8 PMC7265389

[CR24] King CE, Griffin WC, Luderman LN, Kates MM, McGinty JF, Becker HC (2017) Oxytocin reduces ethanol Self-Administration in mice. Alcohol Clin Exp Res 41:955–964 28212464 10.1111/acer.13359 PMC5404956

[CR25] Kirsch P, Esslinger C, Chen Q, Mier D, Lis S, Siddhanti S, Gruppe H, Mattay VS, Gallhofer B, Meyer-Lindenberg A (2005) Oxytocin modulates neural circuitry for social cognition and fear in humans. J Neurosci 25:11489–11493 16339042 10.1523/JNEUROSCI.3984-05.2005 PMC6725903

[CR26] Koob GF (2021) Drug addiction: hyperkatifeia/negative reinforcement as a framework for medications development. Pharmacol Rev 73:163–201 33318153 10.1124/pharmrev.120.000083 PMC7770492

[CR27] Koob GF, Vendruscolo L (2023) Theoretical frameworks and mechanistic aspects of alcohol addiction: alcohol addiction as a reward deficit/stress surfeit disorder. Springer 10.1007/7854_2023_424 37421551

[CR28] Koob GF, Volkow ND (2010) Neurocircuitry of addiction. Neuropsychopharmacology 35:217–238 19710631 10.1038/npp.2009.110 PMC2805560

[CR29] Kwako LE, Schwandt ML, Sells JR, Ramchandani VA, Hommer DW, George DT, Sinha R, Heilig M (2015) Methods for inducing alcohol craving in individuals with co-morbid alcohol dependence and posttraumatic stress disorder: behavioral and physiological outcomes. Addict Biol 20:733–746 24806358 10.1111/adb.12150 PMC4224641

[CR30] Laux L, Glanzmann P, Schaffner P, Spielberger C (1981) Das State-Trait-Angstinventar (Testmappe Mit Handanweisung, Fragebogen STAI-G form X 1 und Fragebogen STAI-G form X 2). Beltz, Weinheim

[CR31] Lieberz J, Scheele D, Spengler FB, Matheisen T, Schneider L, Stoffel-Wagner B, Kinfe TM, Hurlemann R (2020) Kinetics of Oxytocin effects on amygdala and striatal reactivity vary between women and men. Neuropsychopharmacology 45:1134–1140 31785587 10.1038/s41386-019-0582-6 PMC7235226

[CR32] Lim C-Y, In J (2021) Considerations for crossover design in clinical study. Korean J Anesthesiology 74:293 10.4097/kja.21165 PMC8342834 34344139

[CR33] Litten RZ, Falk DE, Ryan ML, Fertig J, Leggio L (2018) Advances in Pharmacotherapy Development: human clinical studies. In: Grant KA, Loving DM (eds) The Neuropharmacology of Alcohol. Springer International Publishing, Cham, pp 579–613 10.1007/164_2017_79 29294197

[CR34] MacFadyen K, Loveless R, DeLucca B, Wardley K, Deogan S, Thomas C, Peris J (2016) Peripheral Oxytocin administration reduces ethanol consumption in rats. Pharmacol Biochem Behav 140:27–32 26519603 10.1016/j.pbb.2015.10.014 PMC4859306

[CR35] Mann KA, K (2000) Die OCDS-G: psychometrische Kennwerte der Deutschen version der obsessive compulsive drinking scale. Sucht 46:10

[CR36] Melby K, Grawe RW, Aamo TO, Salvesen O, Spigset O (2019) Effect of intranasal Oxytocin on alcohol withdrawal syndrome: A randomized placebo-controlled double-blind clinical trial. Drug Alcohol Depend 197:95–101 30784955 10.1016/j.drugalcdep.2019.01.003

[CR37] Melby K, Grawe RW, Aamo TO, Skovlund E, Spigset O (2021) Efficacy of Self-Administered intranasal Oxytocin on alcohol use and craving after detoxification in patients with alcohol dependence. A Double-Blind Placebo-Controlled trial. Alcohol Alcohol 56:565–572 33352584 10.1093/alcalc/agaa133 PMC8406061

[CR38] Mitchell JM, Arcuni PA, Weinstein D, Woolley JD (2016) Intranasal Oxytocin selectively modulates social perception, craving, and approach behavior in subjects with alcohol use disorder. J Addict Med 10:182–189 27159342 10.1097/ADM.0000000000000213

[CR39] Moeller S, Yacoub E, Olman CA, Auerbach E, Strupp J, Harel N, Ugurbil K (2010) Multiband multislice GE-EPI at 7 Tesla, with 16-fold acceleration using partial parallel imaging with application to high Spatial and Temporal whole-brain fMRI. Magn Reson Med 63:1144–1153 20432285 10.1002/mrm.22361 PMC2906244

[CR40] Monti PM, Binkoff JA, Abrams DB, Zwick WR, Nirenberg TD, Liepman MR (1987) Reactivity of alcoholics and nonalcoholics to drinking cues. J Abnorm Psychol 96:122 3584660 10.1037//0021-843x.96.2.122

[CR41] Oslin DW, Cary M, Slaymaker V, Colleran C, Blow FC (2009) Daily ratings measures of alcohol craving during an inpatient stay define subtypes of alcohol addiction that predict subsequent risk for resumption of drinking. Drug Alcohol Depend 103:131–136 19443131 10.1016/j.drugalcdep.2009.03.009 PMC12272362

[CR42] Pedersen CA, Smedley KL, Leserman J, Jarskog LF, Rau SW, Kampov-Polevoi A, Casey RL, Fender T, Garbutt JC (2013) Intranasal Oxytocin blocks alcohol withdrawal in human subjects. Alcohol Clin Exp Res 37:484–489 23025690 10.1111/j.1530-0277.2012.01958.x PMC3557665

[CR43] Potretzke S, Zhang Y, Li J, Fecteau KM, Erikson DW, Hibert M, Ryabinin AE (2023) Male-selective effects of Oxytocin agonism on alcohol intake: behavioral assessment in socially housed prairie voles and involvement of RAGE. Neuropsychopharmacology: Official Publication Am Coll Neuropsychopharmacol 48:920–928 10.1038/s41386-022-01490-3 PMC10156683 36369481

[CR44] Quintana DS, Guastella AJ (2020) An allostatic theory of Oxytocin. Trends Cogn Sci 24:515–528 32360118 10.1016/j.tics.2020.03.008

[CR45] Quintana DS, Lischke A, Grace S, Scheele D, Ma Y, Becker B (2021) Advances in the field of intranasal Oxytocin research: lessons learned and future directions for clinical research. Mol Psychiatry 26:80–91 32807845 10.1038/s41380-020-00864-7 PMC7815514

[CR46] Roesner S, Hackl-Herrwerth A, Leucht S, Vecchi S, Srisurapanont M, Soyka M (2010) Opioid antagonists for alcohol dependence. Cochrane Database Syst Rev 12: CD001867 10.1002/14651858.CD001867.pub3 PMC13590218 21154349

[CR47] Schacht JP, Anton RF, Myrick H (2013) Functional neuroimaging studies of alcohol cue reactivity: a quantitative meta-analysis and systematic review. Addict Biol 18:121–133 22574861 10.1111/j.1369-1600.2012.00464.x PMC3419322

[CR48] Setsompop K, Gagoski BA, Polimeni JR, Witzel T, Wedeen VJ, Wald LL (2012) Blipped-controlled aliasing in parallel imaging for simultaneous multislice echo planar imaging with reduced g-factor penalty. Magn Reson Med 67:1210–1224 21858868 10.1002/mrm.23097 PMC3323676

[CR49] Spanagel R, Bach P, Banaschewski T, Beck A, Bermpohl F, Bernardi RE, Beste C, Deserno L, Durstewitz D, Ebner-Priemer U (2024) The recode addiction research consortium: losing and regaining control over drug intake—Findings and future perspectives. Addict Biol 29:e13419 38949209 10.1111/adb.13419 PMC11215792

[CR50] Stevenson JR, Wenner SM, Freestone DM, Romaine CC, Parian MC, Christian SM, Bohidar AE, Ndem JR, Vogel IR, O’Kane CM (2017) Oxytocin reduces alcohol consumption in prairie voles. Physiol Behav 179:411–421 28716609 10.1016/j.physbeh.2017.07.021

[CR51] Suzuki S, Mell MM, O’Malley SS, Krystal JH, Anticevic A, Kober H (2020) Regulation of craving and negative emotion in alcohol use disorder. Biol Psychiatry: Cogn Neurosci Neuroimaging 5:239–250 31892465 10.1016/j.bpsc.2019.10.005 PMC7010564

[CR52] Tully J, Gabay AS, Brown D, Murphy DGM, Blackwood N (2018) The effect of intranasal Oxytocin on neural response to facial emotions in healthy adults as measured by functional MRI: A systematic review. Psychiatry Res 272:17–29 10.1016/j.pscychresns.2017.11.017 PMC6562202 29272737

[CR53] Tunstall BJ, Kirson D, Zallar LJ, McConnell SA, Vendruscolo JCM, Ho CP, Oleata CS, Khom S, Manning M, Lee MR, Leggio L, Koob GF, Roberto M, Vendruscolo LF (2019) Oxytocin blocks enhanced motivation for alcohol in alcohol dependence and blocks alcohol effects on GABAergic transmission in the central amygdala. PLoS Biol 17:e2006421 30990816 10.1371/journal.pbio.2006421 PMC6467366

[CR54] Vetter S, Reichl M, Sirignano L, Grinevich V, Koopmann A, Spanagel R, Kiefer F, Sommer W, Bach P (2025) Oxytocin increases neural cue-reactivity but modulates cue-induced craving differently in males and females with alcohol use disorder - results of a randomized controlled cross-over trial. osf.io/q8jak_v1 10.31219/osf.io/q8jak_v1 10.1007/s00213-025-06779-x PMC12380973 40232386

[CR55] Vollstadt-Klein S, Loeber S, Richter A, Kirsch M, Bach P, von der Goltz C, Hermann D, Mann K, Kiefer F (2012) Validating incentive salience with functional magnetic resonance imaging: association between mesolimbic cue reactivity and attentional bias in alcohol-dependent patients. Addict Biol 17:807–816 21790907 10.1111/j.1369-1600.2011.00352.x

[CR56] Wellek S, Blettner M (2012) On the proper use of the crossover design in clinical trials: part 18 of a series on evaluation of scientific publications. Dtsch Arztebl Int 109:276–281 22567063 10.3238/arztebl.2012.0276 PMC3345345

[CR57] Witkiewitz K, Villarroel NA (2009) Dynamic association between negative affect and alcohol lapses following alcohol treatment. J Consult Clin Psychol 77:633 19634957 10.1037/a0015647 PMC2911993

[CR58] World Health Organization (2018) Global status report on alcohol and health 2018. World Health Organization.

