# Supplementary material for: Intranasal oxytocin blunts amygdala response to negative affective stimuli in males and females with alcohol use disorder: a randomized controlled cross-over trial
Source: Psychopharmacology (Berl). 2025 Mar 31;242(9):1995–2007. doi: 10.1007/s00213-025-06779-x (PMC12380973; doi:10.1007/s00213-025-06779-x)

SUPPLEMENTARY INFORMATION

Intranasal oxytocin blunts amygdala response to negative affective stimuli in males and females with alcohol use disorder: a randomized controlled cross-over trial

in *Psychopharmacology*

Sina Vetter MSc[1,2], Sophia Schnabel[1,2], Matthias Reichl[1,2], Lea Sirignano MSc[4], Valery Grinevich PhD[2,5], Anne Koopmann MD[1,3], Rainer Spanagel PhD[2,6], Falk Kiefer MD[1,2,3]*, Wolfgang Sommer MD[2,6,7]*, Patrick Bach MD PhD[1,2,3]*

[1] Department of Addictive Behavior and Addiction Medicine, Central Institute of Mental Health, Medical Faculty Mannheim, University of Heidelberg, Mannheim, Germany
[2] German Center for Mental Health (DZPG) - Partner Site Mannheim-Heidelberg-Ulm, Germany
[3] Feuerlein Center on Translational Addiction Medicine (FCTS), University of Heidelberg, Germany
[4] Department of Genetic Epidemiology in Psychiatry, Central Institute of Mental Health, Medical Faculty Mannheim, University of Heidelberg, Mannheim, Germany
[5] Department of Neuropeptide Research in Psychiatry, Central Institute of Mental Health, Medical Faculty Mannheim, University of Heidelberg, J5, 68159 Mannheim, Germany
[6] Institute of Psychopharmacology, Central Institute of Mental Health, Medical faculty Mannheim, University of Heidelberg, Mannheim, Germany
[7] Bethanien Hospital for Psychiatry, Greifswald, Germany
* = these authors contributed equally

Corresponding author:
Sina Vetter, MSc
Department of Addictive Behavior and Addiction Medicine
Central Institute of Mental Health
J5 / 68159 Mannheim, Germany
Tel.: +49 621 1703 3922
E-mail: sina.vetter@zi-mannheim.de

Content:

- **Supplementary Tables**
  - **Supplementary Table S1**. Demographic data and substance use patterns of all randomized individuals (N = 23).
- **Supplementary Figures**
  - **Supplementary Figure S1**. Depiction of the positive correlation between activation in the right amygdala and alcohol craving measured by the Alcohol Urge Questionnaire (AUQ) in A] female compared to B] male individuals with Alcohol Use Disorder.

Supplementary Tables

Supplementary Table S1. Demographic data and substance use patterns of all randomized individuals (*N* = 23).

	**Individuals with AUD** ***N* = 23**
Demographical variables	
Sex (female/male)	12/11
Age [years; mean (SD)]	45.87 (11.88)
Substance use characteristics	
Number of AUD criteria last 12 months	3.52 (2.13)
ADS [total score; mean (SD)]	7.04 (4.73)
Smoker (yes/no)	4/19
FTND [total score; mean (SD)]	1.53 (2.83)

Note. AUD = Alcohol Use Disorder, ADS = Alcohol Dependence Scale, FTND = Fagerstrøm Test for Nicotine Dependence

Supplementary Figures

Supplementary Figure S1. Depiction of the positive correlation between activation in the right amygdala and alcohol craving measured by the Alcohol Urge Questionnaire (AUQ) in A] female compared to B] male individuals with Alcohol Use Disorder.

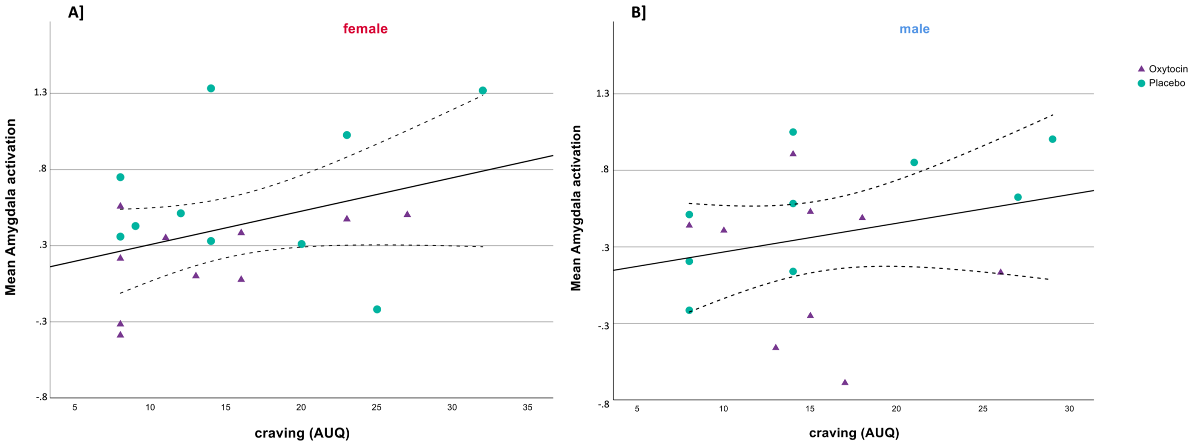

Supplement: Supplementary file 1 — Supplementary Material 1 [file 213_2025_6779_MOESM1_ESM.pdf]
